# Open science priorities for rigorous nature-based climate solutions

**DOI:** 10.1371/journal.pbio.3001929

**Published:** 2022-12-12

**Authors:** William R. L. Anderegg, Anna T. Trugman, Jonathan Wang, Chao Wu

**Affiliations:** 1 Wilkes Center for Climate Science and Policy, University of Utah, Salt Lake City, Utah, United States of America; 2 School of Biological Sciences, University of Utah, Salt Lake City, Utah, United States of America; 3 Department of Geography, University of California Santa Barbara, Goleta, California, United States of America; 4 Department of Earth System Science, University of California Irvine, Irvine, California, United States of America

## Abstract

Open data and science are crucial for robust and scalable nature-based climate solution efforts. This Perspective discusses key opportunities, challenges, and needs for open science to inform these efforts.

## Teaser

Nature-based climate solutions (NbCS) hold promise, but must be based on the best available science to be successful. We outline key ingredients of open data and science crucial for robust and scalable nature-based climate solutions efforts, as an urgent call to action for academic researchers, nongovernmental organizations, government agencies, and private companies.

**Fig 1:**
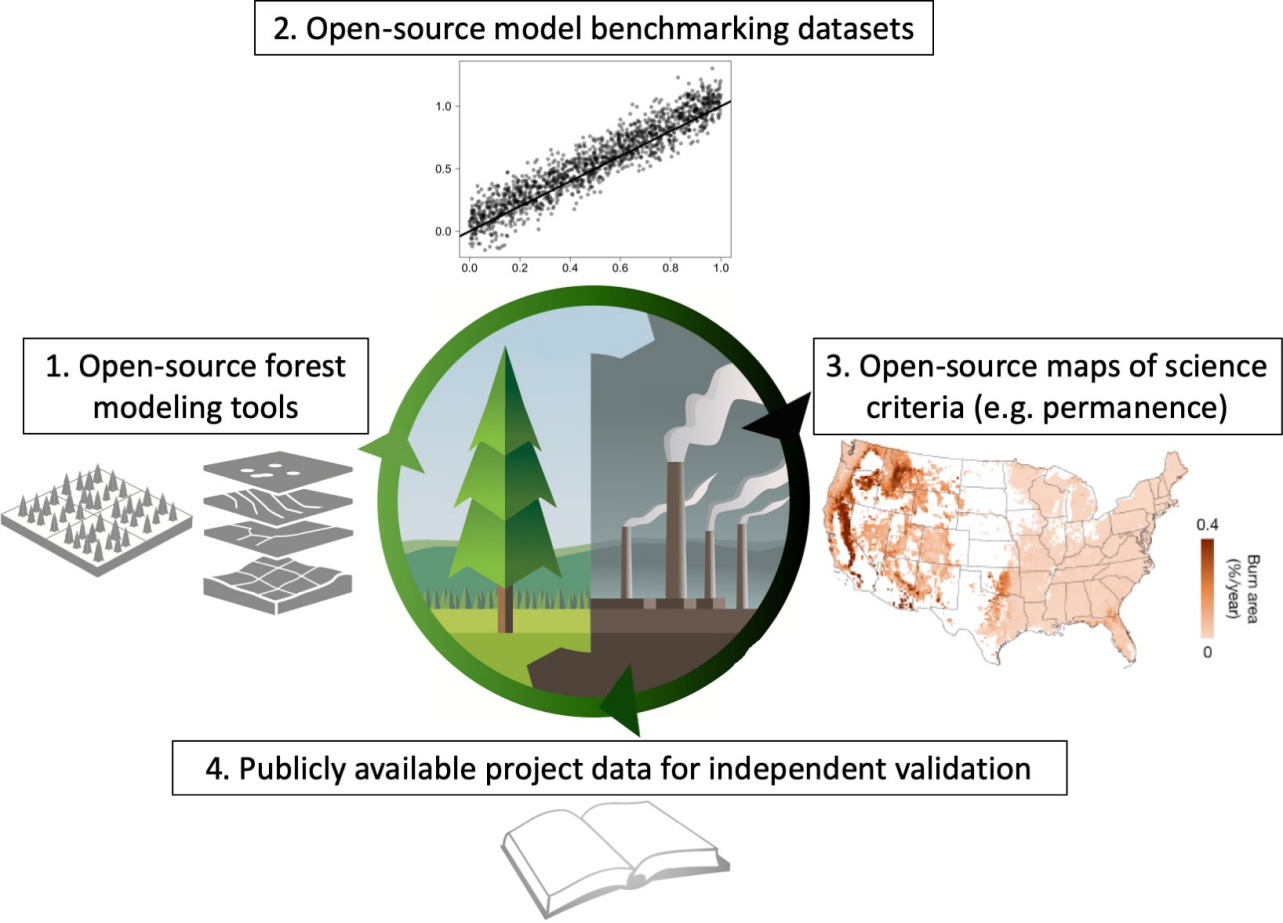
Open science needs for forest nature-based climate solutions. A broad range of open tools and data are crucial for supporting, informing, and evaluating nature-based climate solution efforts broadly. Clip art illustration by David Meikle or from WikiMedia Commons. Geographic basemap from Natural Earth (https://www.naturalearthdata.com/downloads/10m-cultural-vectors/) and burn area model from ref [12].

Nature-based climate solutions (NbCS) are growing rapidly around the globe and can potentially help stabilize climate if paired with aggressive climate mitigation [1]. However, many current NbCS efforts are often not based on rigorous scientific data or tools, and the assumptions, models, and data underlying broad swaths of carbon offsets markets, currently worth multiple billions of dollars and likely to grow substantially in coming years, are frequently opaque, not replicable, and not comparable [2,3]. Open-source data and tools must form the foundation of NbCS efforts to ensure that they are robust, credible, scalable, and independently verifiable. This is an enormous and urgent call to action for academic researchers, nongovernmental organizations, government agencies, and private companies. We posit here at least 4 key ingredients of open science needed in NbCS efforts around the globe (Fig 1). We focus primarily on forest-related NbCS efforts, though similar principles apply across all NbCS sectors/systems.

First, the availability of open-source, standardized modeling tools and model training are critical to the success of NbCS. As one example, the main modeling tool utilized in many current United States forest offsets programs is the Forest Vegetation Simulator (FVS), which simulates the effects of forest vegetation change in response to management, disturbances, and natural succession. FVS is used to calculate carbon crediting for a given NbCS project due to practices such as improved forest management. One major limitation of FVS in the context of NbCS is the lack of climate sensitivity underlying model projections for forest dynamics. In other words, future growth, mortality, demography, and biomass are not influenced by changes in climate variables such as temperature or precipitation. Moving forward, it is possible to use state-of-the-science demographic models that have a realistic representation of plant physiological parameters, ecological dynamics, and climate feedbacks [4]. Within the category of demographic models, there are a number of individual models with different representations of plant physiology and ecological processes. To be successfully utilized in an NbCS framework, it is imperative that the (i) model code; (ii) model input parameters that reflect plant physiological and ecological processes; and (iii) meteorological forcing datasets for the model tools are open-source and standardized across NbCS projects. We recommend that selection criteria for a standardized set of models involve rigorous validation using ground and remote sensing products and that the models be assessed for performance using the International Land Model Benchmarking (ILAMB; [5]) or a similar model assessment tool. Models or other tools that perform poorly during validation should be excluded.

Second, open-source datasets for monitoring, reporting, and validation are crucial for quantifying when, where, and how much ecosystem properties have changed and for assessing the efficacy of NbCS. In the past, satellite-based datasets have been used for identifying forest loss in support of tropical forest protection programs such as Reducing Emissions from Deforestation and forest Degradation (REDD+; [6]). However, new remote sensing platforms can provide a more nuanced, detailed view into ecosystem dynamics that reflect the impacts of human intervention and climate change at scale. For example, lidar instruments such as GEDI can map aboveground biomass density at regional scales and high resolutions [7], allowing quantification of carbon sequestration across space and time. In addition, publicly accessible field data, such as the US Forest Inventory and Analysis (FIA) dataset available through the R package rFIA [8], will be key in ground-truthing satellite-based datasets and for estimating forest attributes not accessible via remote sensing. Thus, we recommend that open-source algorithms and high-resolution, spatially complete maps are used to enable NbCS stakeholders to quantify the impact of individual projects and regional programs with transparency, credibility, and consistency across a range of jurisdictions and ecosystem types.

Third, more open-source maps and tools to quantify and extract (i) co-benefits from NbCS efforts; (ii) climate and anthropogenic risks to permanence/durability; (iii) additionality; (iv) leakage; and (v) biophysical (net climate) impacts of NbCS efforts are urgently needed. Some initial datasets are available in certain regions, such as ref [9] for net climate impacts, that could be better utilized but these 5 areas need substantial interdisciplinary research efforts. Most work to date has focused on the potential of NbCS, but a wide swath of major limitations could constrain the effectiveness and deployment of NbCS efforts [10]. For example, maps of the extent to which climate change and climate-related risks (e.g., wildfires, climate stress/drought, pests and pathogens, winds, heat waves, and ice/snow) may fundamentally undermine NbCS carbon storage permanence and durability (usually for 100 years or longer) is a major scientific need. Many NbCS protocols rely on self-insurance programs for climate risks (e.g., a “buffer pool” of credits), yet recent work shows that these 100-year buffer pools may be substantially undercapitalized [2,11,12]. Open-source maps and tools of biophysical, economic, land-use, and operational constraints in determining the realistic potential of NbCS are also urgently needed [9,10].

Finally, open data reporting from NbCS efforts and projects are crucial for enabling external and independent validation efforts. For example, data reporting requirements from NbCS projects should include project coordinates, geographic shapefiles, and species composition/stand structure. Data, assumptions, and parameters used to run model simulations for projects of project baselines and management decisions are also key to report and standardize. Standardized databases of projects across different regions and protocols can play a key role in reporting and verification of claims by independent analyses.

Among many others, these elements of open science and data are crucial ingredients in the success of NbCS efforts to mitigate and adapt to climate change. Processes that ensure frequent dialogue and input between NbCS stakeholders and the scientific community, such that key scientific needs are communicated by stakeholders and current scientific understanding is folded into NbCS policies and protocols, will also be important. A broad range of communities and stakeholders have critical roles to play in ensuring transparency and rigor in helping NbCS deliver benefits to ecosystems, society, and the climate.

## References

[1] Matthews HD, Zickfeld K, Dickau M, MacIsaac AJ, Mathesius S, Nzotungicimpaye C-M, et al. Temporary nature-based carbon removal can lower peak warming in a well-below 2° C scenario. Commun Earth Environ. 2022;3:1–8.

[2] Anderegg WR, Trugman AT, Badgley G, Anderson CM, Bartuska A, Ciais P, et al. Climate-driven risks to the climate mitigation potential of forests. Science. 2020;368. 10.1126/science.aaz7005 32554569

[3] Coffield SR, Vo CD, Wang JA, Badgley G, Goulden ML, Cullenward D, et al. Using remote sensing to quantify the additional climate benefits of California forest carbon offset projects. Glob Chang Biol. 2022. doi: 10.1111/gcb.16380 36093912 PMC9826164

[4] Fisher RA, Koven CD, Anderegg WR, Christoffersen BO, Dietze MC, Farrior CE, et al. Vegetation demographics in Earth System Models: A review of progress and priorities. Glob Chang Biol. 2018;24:35–54. doi: 10.1111/gcb.13910 28921829

[5] Collier N, Hoffman FM, Lawrence DM, Keppel-Aleks G, Koven CD, Riley WJ, et al. The International Land Model Benchmarking (ILAMB) system: design, theory, and implementation. J Adv Model Earth Syst. 2018;10:2731–2754.

[6] Goetz SJ, Hansen M, Houghton RA, Walker W, Laporte N, Busch J. Measurement and monitoring needs, capabilities and potential for addressing reduced emissions from deforestation and forest degradation under REDD+. Environ Res Lett. 2015;10:123001.

[7] Dubayah R, Armston J, Healey SP, Bruening JM, Patterson PL, Kellner JR, et al. GEDI launches a new era of biomass inference from space. Environ Res Lett. 2022;17:095001.

[8] Stanke H, Finley AO, Weed AS, Walters BF, Domke GM. rFIA: An R package for estimation of forest attributes with the US Forest Inventory and Analysis database. Environ Model Software. 2020;127:104664.

[9] Williams CA, Gu H, Jiao T. Climate impacts of US forest loss span net warming to net cooling. Sci Adv. 2021;7:eaax8859. 33579704 10.1126/sciadv.aax8859 PMC7880589

[10] Zeng Y, Sarira TV, Carrasco LR, Chong KY, Friess DA, Lee JSH, et al. Economic and social constraints on reforestation for climate mitigation in Southeast Asia. Nat Clim Change. 2020;10:842–844.

[11] Badgley G, Chay F, Chegwidden OS, Hamman JJ, Freeman J, Cullenward D. California’s forest carbon offsets buffer pool is severely undercapitalized. Front For Glob Change. 2022. 10.3389/ffgc.2022.930426

[12] Anderegg WR, Chegwidden OS, Badgley G, Trugman AT, Cullenward D, Abatzoglou JT, et al. Future climate risks from stress, insects and fire across US forests. Ecol Lett. 2022;25:1510–1520. doi: 10.1111/ele.14018 35546256 PMC9321543

